# Associations between Children’s Genetic Susceptibility to Obesity, Infant’s Appetite and Parental Feeding Practices in Toddlerhood

**DOI:** 10.3390/nu13051468

**Published:** 2021-04-26

**Authors:** Claire Guivarch, Marie-Aline Charles, Anne Forhan, Ken K. Ong, Barbara Heude, Blandine de Lauzon-Guillain

**Affiliations:** 1 Université de Paris, CRESS, INSERM, INRAE, F-75004 Paris, France; marie-aline.charles@inserm.fr (M.-A.C.); anne.forhan@inserm.fr (A.F.); barbara.heude@inserm.fr (B.H.); blandine.delauzon@inserm.fr (B.d.L.-G.); 2 Unité Mixte Inserm-Ined-EFS ELFE, Ined, F-75020 Paris, France; 3 MRC Epidemiology Unit and Department of Paediatrics, Institute of Metabolic Science, University of Cambridge, Cambridge CB2 0QQ, UK; Ken.Ong@mrc-epid.cam.ac.uk

**Keywords:** parental feeding practices, genetic susceptibility to obesity, eating behavior, birth cohort

## Abstract

Previous findings suggest that parental feeding practices may adapt to children’s eating behavior and sex, but few studies assessed these associations in toddlerhood. We aimed to study the associations between infant’s appetite or children’s genetic susceptibility to obesity and parental feeding practices. We assessed infant’s appetite (three-category indicator: low, normal or high appetite, labelled 4-to-24-month appetite) and calculated a combined obesity risk-allele score (genetic risk score of body mass index (BMI-GRS)) in a longitudinal study of respectively 1358 and 932 children from the EDEN cohort. Parental feeding practices were assessed at 2-year-follow-up by the CFPQ. Three of the five tested scores were used as continuous variables; others were considered as binary variables, according to the median. Associations between infant’s appetite or child’s BMI-GRS and parental feeding practices were assessed by linear and logistic regression models, stratified on child’s sex if interactions were significant. 4-to-24-month appetite was positively associated with restrictive feeding practices among boys and girls. Among boys, high compared to normal 4-to-24-month appetite was associated with higher use of food to regulate child’s emotions (OR [95% CI] = 2.24 [1.36; 3.68]). Child’s BMI-GRS was not related to parental feeding practices. Parental feeding practices may adapt to parental perception of infant’s appetite and child’s sex.

## 1. Introduction

Childhood overweight and obesity are a major public health challenge of this century, affecting an estimated 38.2 million children under 5 years old worldwide in 2019 [1]. Childhood obesity is associated with short- and long-term adverse outcomes, such as adulthood obesity and cardio-metabolic disorders [2]. Obesity is mostly caused by interactions between genetic susceptibility and obesogenic environment [3,4]. A great number of the genes identified by genome-wide association study of obesity and also those from studies of monogenic forms of severe childhood obesity appear to be involved in the central regulation of food intake [5]. Moreover, several studies have shown that genetic susceptibility to obesity affects infant [6] and child’s appetite [4,7] and that early appetite is related to the child’s weight status later in childhood [8,9,10].

According to the Developmental Origins of Health and Disease concept, early childhood is a vulnerability window: the first years of life are characterized by a rapid change in infant feeding from milk to solid foods that will lay the foundation for future eating habits and behaviors [11,12]. Parents play a key role in the development of healthy eating habits and eating behavior in childhood in that they decide which foods the child is offered as well as the portion size, feeding time and meal environment [12,13]. Moreover, parents play a model role [14,15]. The influence of parents on their child’s eating behavior development starts in the first weeks of life with breastfeeding. Indeed, previous studies suggest that breastfeeding is positively associated with infant’s self-regulation capacities [12] and is also related to child’s eating behavior [16] and parental feeding practices [17]. Other studies suggest that parental feeding practices may differ according to child’s sex and that, for the same parental feeding practice, child’s response may depend on their sex [18,19]. Moreover, several cross-sectional studies have found that coercive parental feeding practices, such as restriction or pressure to eat, are related to child’s weight status (e.g., parental restrictive feeding practices are associated to higher child’s BMI, whereas parental pressure to eat is associated to lower child’s BMI) [20,21,22], child’s intake (e.g., parental restrictive practices are associated with increased child’s energy intake) [23,24,25] or eating behavior (e.g., parental pressure to eat may enhance food dislikes) [26,27]. Some longitudinal studies found that coercive feeding practices led to lower childhood BMI [28,29]. Recent longitudinal studies have highlighted that associations between child’s weight status and eating behavior and parental feeding practices could be more complex, non-linear and bidirectional [13,30,31,32]. They have especially suggested that parents may adapt their feeding practices to the appetite of their child (i.e., using restriction if the infant’s appetite is considered high) [31] and to the child’s weight status [33,34,35,36]. The child’s genetic susceptibility to obesity appeared positively related to infant’s appetite [6] and to appetitive traits in childhood [7,37], but a recent review stated that it remains unclear how genetic and parental feeding practices interact to influence child’s appetite [38].

In this context, we aimed to study the associations between infant’s appetite or child’s genetic susceptibility to obesity and parental feeding practices in toddlerhood with a longitudinal design. We hypothesized that (1) infants perceived as always asking for food during first years of life may lead to higher parental restrictive feeding practices and higher parental use of food for non-nutritional purposes (i.e., using food to manage infant’s emotions or as a reward); (2) infants perceived as having low appetite during the first years of life may lead to greater parental pressure to eat, and (3) a higher child’s genetic risk score of body mass index (BMI-GRS) may lead to higher use of restrictive parental feeding practices and lower use of pressure to eat.

## 2. Materials and Methods

### 2.1. Study Population

The EDEN mother-child study is a prospective cohort aiming at assessing prenatal and postnatal determinants of childhood growth, development and health [39]. Briefly, 2002 pregnant women under 24 weeks’ amenorrhea were recruited from 2003 to 2006 in two French university hospitals, in Poitiers and Nancy. Exclusion criteria were multiple pregnancies, known diabetes before pregnancy, illiteracy and planning to move outside the region in the next 3 years. This study was approved by the ethics research committee of Bicêtre hospital (ID 0270 of 12 December 2002) and by the National Commission on Informatics and Liberty (CNIL, ID 902267 of 12 December 2002). Written consent was obtained from both parents.

Data collected during pregnancy and at birth included sociodemographic variables, maternal smoking and newborn characteristics (sex, gestational age, birthweight). At 4, 8, 12 and 24 months after birth, mothers completed mailed questionnaires that provided detailed information on their feeding practices.

### 2.2. Infant’s Appetite

At ages 4, 8, 12 and 24 months, maternal perception of infant’s appetite was assessed with a single item, translated as “Usually, would you say that your baby: (1) is always hungry or demanding to feed? (2) demands to feed the same as other babies of the same age? (3) needs to be stimulated to eat (at 4, 8 and 12 months) or is not often hungry (at 24 months)?” High appetite was defined by the category “is always hungry or demanding to feed”, normal appetite by the second category “demands to feed the same as other babies of the same age” and low appetite by the third category “needs to be stimulated to eat/not often hungry”. Using the four age-specific variables, infants were classified in the “low appetite” category when parents reported a low appetite at least once up to 24 months and never reported a high appetite during this period. Infants were classified in the “high appetite” category when parents reported a high appetite at least once up to 24 months and never reported a low appetite during this period. All other infants were classified in the “normal appetite” category.

### 2.3. Children’s Genetic Susceptibility to Obesity

DNA was extracted from cord-blood samples collected at birth [39]. As previously described, genotyping candidate single nucleotide polymorphisms (SNPs) was conducted at the Medical Research Council Epidemiology Unit, Cambridge (iPLEX platform; Sequenom) [40]. Among the 32 loci identified by Speliotes et al. as having genome-wide significant associations with BMI in adults [41], in the present study, we considered the 16 SNPs also showing associations with childhood BMI in that original report [41] or in subsequent data [42], as in previous studies [6]. Briefly, a combined obesity risk-allele score, indicating genetic susceptibility to obesity (BMI-GRS), was calculated for each included infant as the sum of risk alleles (0, 1 or 2) associated with higher BMI across the 16 SNPs. In the present study, the score ranged from 5 to 22 from a possible range of 0 to 32.

### 2.4. Parental Feeding Practices

At the 2-year follow-up, parental feeding practices were evaluated by using the Comprehensive Feeding Practices Questionnaire (CFPQ) [43] translated in French and validated in French children [44]. In the present analysis, five scales of the questionnaire were used: restriction for health (4 items, e.g., if I did not guide or regulate my child’s eating, s/he would eat too much of his/her favourite foods, Cronbach’s α = 0.79), restriction for weight (4 items, e.g., I encourage my child to eat less so he/she won’t get fat, Cronbach’s α = 0.67), pressure to eat (3 items, e.g., my child should always eat all of the food on his/her plate, Cronbach’s α = 0.59), using food as a reward (3 items, e.g., I offer my child his/her favourite foods in exchange for good behaviour, Cronbach’s α = 0.45) and using food to regulate the child’s emotions (3 items, e.g., do you give this child something to eat or drink if s/he is upset even if you think s/he is not hungry?, Cronbach’s α = 0.66). Each item is associated with a score between 1 (never or disagree) and 5 (always or agree). Scores of coercive parental feeding practices (restriction for health, restriction for weight and pressure to eat) were considered as continuous variables. Because scores were not normally distributed and transformations tested did not help to reach normality, parental feeding practices of using food as a reward or to regulate the child’s emotions were considered as binary variables, according to the median in our sample. “Normal use” of a specific parental feeding practice was defined by a score below the median and “high use” by a score equal to or above the median.

### 2.5. Potential Confounders

The maternal characteristics were collected at the maternity ward and included maternal age at delivery (years), primiparity (yes/no), maternal education level (<high school diploma, high school diploma, 2-year university degree and 5-year university degree), household income (≤€1500, €1501 to €2300, €2301 to €3000 and >€3000) and smoking status during pregnancy (no smoker/ smoker). The child’s characteristics were collected at birth and during the first year: sex, birth weight (kg), any breastfeeding duration (<1 month, 1 to <4 months and at least 4 months) and age at complementary food introduction (months). At each clinical examination (birth, 1, 3 and 5 years), the child’s weight and length were measured. At each follow-up (4, 8 and 12 months, 2, 3, 4 and 5 years), weight and length data were collected from self-administered questionnaires and clinical visits when reported by health professionals in the child’s health booklet. Individual growth curves for weight and length were predicted by using the Jenss growth curve model as previously described [45]. This method allows for calculating parameters for individual growth patterns, such as weight, length and body mass index (BMI). In the present study, we used the WHO weight-for-length z-score at 2 years as a covariate in sensitivity models.

### 2.6. Sample Selection

**Figure 1:**
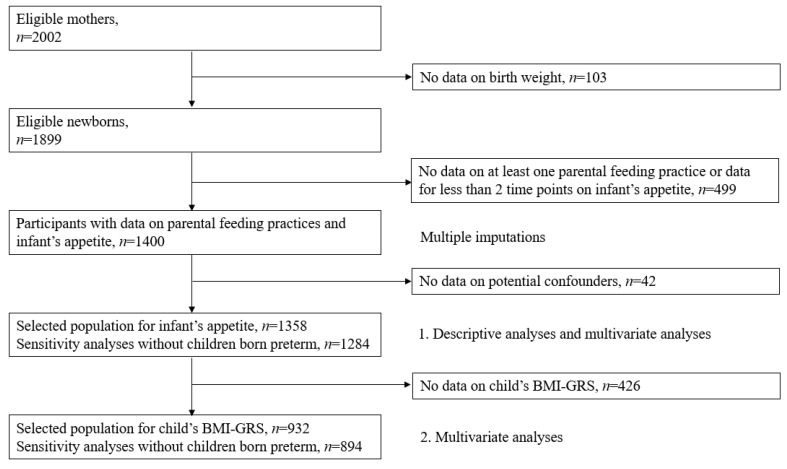
Flow of participants in the study.

Of the 2002 women recruited, 76 were excluded because they left the study before or at the time of delivery; 24 because of miscarriages, intrauterine death or discontinuation of pregnancy for medical reasons; and nine because they delivered outside the study hospitals. Data on birthweight were available for 1899 newborns. Infants with missing data on at least one parental feeding practice or less than 2 time points for infant’s appetite assessment (*n* = 499) and on potential confounders (*n* = 42) were then excluded (Figure 1). These exclusions lead to a sample of 1358 infants for complete-case analysis of the association between infant’s appetite and parental feeding practices.

Children with missing data on child’s BMI-GRS were excluded for analyses involving BMI-GRS, leading to a sample of 932 infants.

### 2.7. Statistical Analyses

#### 2.7.1. Main Analyses

Comparisons between included and excluded populations were assessed by chi-square and Student t-tests. Bivariate analyses between infant’s appetite or child’s BMI-GRS and parental feeding practices involved unadjusted linear and logistic regression models. Associations between infant’s appetite or child’s BMI-GRS and parental feeding practices were tested with linear regression models for coercive parental feeding practices and with logistic regression models for parental use of food for non-nutritional purposes. Analyses were run separately for each outcome. We tested the interaction between child’s sex and infant’s appetite for each parental feeding practice. Significant interactions were observed for the following feeding practices: restriction for health (*p* = 0.003), restriction for weight (*p* = 0.0004), emotional feeding (*p* = 0.02) and the interaction was almost significant for food as a reward (*p* = 0.08). Thus, analyses were performed separately among girls and boys for these feeding practices. As no significant interaction was found between child’s BMI-GRS and child’s sex (all *p* > 0.2), the associations between child’s BMI-GRS and parental feeding practices were conducted on the whole sample.

For multivariable analyses, potential confounding factors included in the models were identified from the literature and selected by using the Directed Acyclic Graphs method. Then, models used to test the association between child’s BMI-GRS and parental feeding practices were adjusted only for study center, whereas models used to test the associations between infant’s appetite and parental feeding practices were adjusted for study center as well as for maternal characteristics (age at delivery, primiparity, education level, household income, smoking status during pregnancy) and child’s characteristics (birth weight, any breastfeeding duration and age at complementary food introduction). Analyses conducted on the whole sample were also adjusted for child’s sex.

#### 2.7.2. Sensitivity Analyses

In the first sensitivity analysis, we excluded infants born before 37 gestational weeks because parental feeding practices may differ for pre-term infants [46], which led to samples of 1284 (*n* = 667 boys and *n* = 617 girls) for infant’s appetite analyses and 894 infants for BMI-GRS analyses. For infant’s appetite analyses, a second sensitivity analysis was performed by further adjusting on child’s WHO weight-for-length z-score at 2 years. In the third sensitivity analysis, we considered 4-to-12-month appetite instead of the 4-to-24-month summary variable, to test the potential influence of children’s current appetite. In another sensitivity analysis, we considered 1-year appetite instead of the 4-to-24-month summary variable, to test the stability of the main findings using a raw variable [6]. Another sensitivity analysis involved a weighted BMI-GRS (in which risk alleles were weighted by their reported effects size on adult BMI) [41,42] instead of the crude BMI-GRS.

We first conducted analyses on complete cases. Then, we used multiple imputations to deal with missing data on exposure variables and potential confounders. Missing data for child’s BMI-GRS were only imputed if maternal BMI-GRS was available. The number of missing data ranged from 0% to 31.1% per variable (Supplementary Table S1). We assumed that data were missing at random and generated five independent datasets with the fully conditional specification method (MI procedure, FCS statement, NIMPUTE option), and then calculated pooled effect estimates (SAS MIANALYSE procedure). Continuous variables were imputed with predictive mean matching, and logistic regressions were used for categorical variables. To generate significance testing of categorical variables, the median of the p-values from the imputed data analyses in each dataset was used, as proposed by Eekhout et al. [47].

Analyses were conducted with SAS v9.4 (SAS Institute, Cary, NC, USA). *p* < 0.05 was considered statistically significant.

## 3. Results

Infants included in and excluded from the present study were similar concerning child’s sex, birth weight and gestational age. However, infants included were breastfed longer and were born to older mothers, with lower BMI, higher education level, higher household income, higher rate of primiparity and lower rate of smoking during pregnancy than those excluded. The maternal and child’s characteristics of the study population, parental feeding practices and infant’s appetite are summarized in Table 1 and Table 2.

### 3.1. Infant’s Appetite and Parental Feeding Practices at 2 Years

In the main adjusted analyses, 4-to-24-month appetite was positively associated to parental restriction for weight among girls (linear trend *p* < 0.001) and boys (linear trend *p* = 0.03) at 2 years (Table 3). Among girls only, 4-to-24-month appetite was positively associated to parental restriction for health (linear trend *p* < 0.001) (Table 3) and to parental use of food as a reward (linear trend *p* = 0.002) at 2 years (Supplementary Table S2). Moreover, low compared to normal 4-to-24-month infant’s appetite was related to higher parental pressure to eat (β [95% CI] = 0.15 [0.01; 0.28]) and high 4-to-24-month infant’s appetite was related to higher parental pressure to eat (β [95% CI] = 0.14 [0.00; 0.28]) at 2 years (Table 3). Among boys only, low compared to normal 4-to-24-month appetite was related to higher use of food as a reward (OR [95% CI] = 2.69 [1.50; 4.81]) and high 4-to-24-month appetite was related to higher use of food as a reward (OR [95% CI] = 1.58 [1.01; 2.49]) (Supplementary Table S2) and to regulate the child’s emotions (OR [95% CI] = 2.24 [1.36; 3.68]) at 2 years (Table 4).

Sensitivity analyses (Table 3 and Table 4, Supplementary Table S2) revealed similar results after excluding infants born before 37 gestational weeks. Findings were similar after further adjustment on child’s 2-year WHO weight-for-length z-score. When considering 4-to-12-month appetite instead of 4-to-24-month appetite, the main finding remained unchanged, but the associations between infant’s appetite and parental pressure to eat were no longer significant. Moreover, among boys, for parental use of food as a reward, the association with low appetite was weakened and became non-significant, but the same tendency was observed. After multiple imputations, similar results were found, except concerning use of food as a reward: among boys, the association with high 4-to-24-month appetite was weakened and no more significant. When we assessed the association between appetite at 1 year and parental feeding practices at 2 years, low 1-year appetite was related to lower parental restriction for health among girls, and lower parental restriction for weight among boys, whereas high 1-year appetite was only related to higher restriction for weight among girls (Supplementary Tables S4 and S5).

### 3.2. Child’s BMI-GRS and Parental Feeding Practices at 2 Years

Child’s BMI-GRS was not related to any of the five parental feeding practices tested in our main analyses. Similar results were found in sensitivity analyses (Table 5 and Table 6, Supplementary Table S3).

## 4. Discussion

In our study, children’s genetic susceptibility to obesity was not associated with any parental feeding practices at 2 years, whereas perceived infant’s appetite was associated with several parental feeding practices at 2 years and differed among child’s sex. Infant’s appetite was positively related to restrictive feeding practices, but associations were stronger among girls than among boys. Moreover, high appetite was related to higher parental use of food to regulate child’s emotions in boys but not in girls. Both low and high infant’s appetite were also associated to higher parental pressure to eat.

During the first year of life, infants have the ability to self-regulate their food intake based on their satiety and hunger cues. Experimental studies have shown that some coercive feeding practices such as parental restriction or pressure to eat may have a counterproductive effect because limiting access to some foods may increase the child’s attraction to the restricted foods [48] or have a negative impact on the child’s food intake self-regulation [49], and parental pressure to eat may enhance food dislikes [26]. Moreover, restrictive parental feeding practices are often considered to lead to overweight and obesity issues in later life, as summarized in literature reviews [33].

More recent studies suggested that the associations between children’s BMI and parental feeding practices are bi-directional [30,32,35,50,51]. Longitudinal studies found that parental restrictive practices are developed in reaction to higher BMI in children, whereas parental pressure to eat develops in reaction to lower BMI in children [30,32,33,34,35], and the influence of children’s BMI on parental feeding practices appeared more important than the influence of parental feeding practices on children’s weight gain [30,32,34,35]. Then, parents may adapt their feeding practices on their perception of the child’s weight status. Nevertheless, most of these studies were based on preschool or school-aged children, and none directly assessed a child’s genetic susceptibility to obesity. In a previous recent study of 10-year-old children from the Twins Early Development Study, higher genetic susceptibility to obesity was associated to higher use of parental restrictive feeding practices and lower use of parental pressure to eat [52]. In the present study, children’s genetic susceptibility was not related to parental feeding practices assessed in toddlerhood. This finding may be due to the BMI-GRS not being related to children’s BMI before age 3 years [42] and then parents not being aware of an increased risk of overweight/obesity for their child. However, we recently highlighted that infant’s appetite may be considered a mediating factor between children’s genetic susceptibility to obesity and children’s BMI [6]. Then, we hypothesized that infant’s appetite could be considered an indicator of children’s obesity risk, more apparent to parents during this early life period. Because an association between child’s BMI-GRS and infant’s appetite appears at an earlier age (age 1 year) [6] than an association between child’s BMI-GRS and child’s BMI (at age 3 years) [42], child’s weight status was not considered in the main analyses. However, in sensitivity analyses, findings were similar after further adjustment on child’s 2-year WHO weight-for-length z-score.

In the present longitudinal study, several associations were highlighted between infant’s appetite and parental feeding practices, some of them depending on child’s sex. This information may be of great importance because parental feeding practices, and children’s appetitive traits appear to be established during the first 2 years of life [31]. Moreover, the early development of unhealthy eating patterns and high BMI in childhood, found to be associated with the score of genetic susceptibility to obesity, could have causal long-term implications on BMI in adulthood [53]. To our knowledge, no study had examined the moderating effect of child’s sex on associations between infant’s appetite and parental feeding practices in toddlerhood. In the present study, we found positive associations between infant’s appetite and parental restriction for weight among boys and girls, supporting the hypothesis that parents are more restrictive with a child perceived as having an important appetite or who is food responsive [54]. We also highlighted that the associations between infant’s appetite and restrictive feeding practices were stronger among girls than among boys, supporting the hypothesis that parents are more likely to be concerned about the weight status of their daughter compared to son [55]. According to literature on the differences of child’s sex on associations between maternal feeding practices and child’s weight status, these results could be explained with societal expectations dealing with child’s weight status: girls should be slim whereas boys are perceived as sturdier if they are larger [18]. As in a previous recent US study including 139 parent-child dyads, high infant’s appetite was related to higher use of pressure to eat in some of our analyses, probably because of parental willingness to increase children’s intake of “healthy foods”, such as fruits and vegetables [56]. We also found that low infant’s appetite was associated with higher use of pressure to eat, suggesting parental willingness to increase their infant’s food intakes if the infant was perceived as having low appetite. Increasing evidence shows a prospective association between parental use of food for non-nutritional purposes in childhood (i.e., as a reward or to manage children’s emotions) and negative impacts on eating behavior and BMI in later life [50,57,58,59]. However, to our knowledge, few studies are available on the influence of children’s appetite on parental use of food for non-nutritional purposes (i.e., using food as a reward or to manage infant’s emotions), with inconsistent results. In fact, some studies found no association between children’s eating behavior and parental use of food for non-nutritional purposes (as a reward or to regulate child’s emotions) [31,57], whereas children’s overeating was found positively associated with using food as a reward [50,59]. This last result is consistent with the associations found among boys, between high infant’s appetite and parental use of food to manage infant’s emotions and with the positive association among girls between infant’s appetite and using food as a reward. Our results suggest that parents use these feeding practices to reward or to calm their child if the infant shows interest in food.

The mothers of the EDEN mother-child cohort have higher socio-economic position and a higher education level than the French population [39], so further studies are needed to assess the validity of our results among lower socio-economic populations. Even though the CFPQ was validated among French children [44], in the present study, parental use of food as a reward had a low Cronbach’s α, limiting the interpretation of the results. Moreover, in the current study, only a specific set of feeding practices were considered: coercive feeding practices and use of food for non-nutritional purposes. Future studies should consider other feeding practices or dimensions of Infant Young Child Feeding, such as breastfeeding and complementary food introduction, to test the potential bi-directional association with infant’s appetite. Infant’s appetite was assessed prospectively from 4 months to 24 months, thus limiting memory bias. If infant’s appetite was assessed by a single item up to 24 months and not a validated scale, due to the absence of a validated questionnaire at the launch of the EDEN mother-child cohort, a similar item was used in previous studies [10], and associations between appetite and growth were similar with this item and with a validated scale [6]. Furthermore, this item reflects maternal perception of infant’s appetite, and further studies are needed to examine maternal ability to read child’s feeding cues and responsive feeding. Moreover, data on infant’s self-perceived regulation were not available in the current study, notably due to the absence of validated questionnaire to assess infant’s self-regulation at the beginning of the EDEN mother-child cohort. Future longitudinal studies should assess the associations between infant’s self-regulation or children’s self-perceived appetite and parental feeding practices.

## 5. Conclusions

In this birth cohort, a BMI-GRS representing children’s genetic susceptibility to obesity was not related to parental feeding practices in toddlerhood. However, in the present study, the associations between infant’s appetite and parental feeding practices were moderated by child’s sex. These results highlighted the need to reinforce parental education concerning feeding practices. Moreover, our results suggest that restriction for weight could be a response to infant’s hungrier appetite among boys and girls. Given the reported moderating effect of restriction on the association between genetic risk of obesity and BMI in adulthood [60], future studies should examine whether restrictive feeding practices in toddlerhood may modulate the association between genetic susceptibility to obesity and BMI later in childhood.

## Figures and Tables

**Table 1:** Characteristics of the study population (*n* = 1358).

			% (n), Mean (SD) or Median (Q1–Q3)
**Maternal Characteristics**		
	Center	
		Poitiers	46.8% (636)
		Nancy	53.2% (722)
	Age at delivery (years)		29.9 (4.7)
	Primiparous		47.2% (641)
	Education level	
		<High school diploma	23.1% (314)
		High school diploma	17.7% (240)
		2 years university degree	23.2% (315)
		5 years university degree	36.0% (489)
	Household income (€/month)	
		≤1500	12.6% (171)
		1501–2300	29.2% (397)
		2301–3000	28.1% (382)
		>3000	30.0% (408)
	Smoker status during pregnancy		22.0% (299)
	BMI before pregnancy (kg/m^2^)		23.1 (4.4)
**Parental Feeding Practices ^a^**		
	Restriction for health		3.4 (1.0)
	Restriction for weight		1.7 (0.6)
	Pressure to eat		2.3 (0.8)
	Food as a reward		1.33 (1.00–1.66)
	Emotional feeding		1.33 (1.00–1.66)
**Child Characteristics**		
	Boys		52.1% (707)
	Birth weight (kg)		3.3 (0.5)
	Gestational age (weeks)		39.2 (1.7)
	Pre-term birth (<37 weeks)		5.4% (74)
	Any breastfeeding duration, months	
		<1	33.3% (452)
		1 to <4	31.0% (421)
		≥4	35.7% (485)
	BMI genetic risk score (0–32 score)		13.7 (2.5)
	WHO weight for length z-score at 2 years		0.2 (1.7)

BMI, body mass index. ^a^ Parental feeding practices were assessed using the Comprehensive Feeding Practices Questionnaire [43] at the 2-year follow-up. Each studied parental feeding practice is associated to a score between 1 and 5. Scores of coercive parental feeding practices (restriction for health, restriction for weight and pressure to eat) were studied as continuous variables. Because scores of parental feeding practices of using food for non-nutritional purposes (as a reward or to regulate child’s emotions) were not normally distributed and those transformations did not help to reach normality, these scores were studied as binary variables, according to the median.

**Table 2:** Description of infant’s appetite from 4 to 24 months.

		4 Months	8 Months	12 Months	24 Months
**Infant appetite**				
	Needs to be stimulated	2.7% (36)	2.7% (36)	4.8% (62)	6.6% (90)
	Normal appetite	93.4% (1239)	95.2% (1252)	92.0% (1188)	88% (1194)
	Always hungry	3.9% (52)	2.1% (27)	3.3% (42)	5.4% (73)
**4-to-24-month appetite**				
	Low appetite	11.1% (151)			
	Normal appetite	77.7% (1055)			
	High appetite	11.2% (152)			

Data are % (n). 4-to-24-month appetite is an indicator of infant’s appetite. Infants were classified in the low appetite category when parents reported a low appetite at least once up to 24 months and never reported a high appetite during this period. Infants were classified in the high appetite category when parents reported a high appetite at least once up to 24 months and never reported a low appetite during this period. All other infants were classified in the normal-appetite category.

**Table 3:** Associations between infant’s appetite (reference = normal appetite) and coercive feeding practices.

			Restriction for Health				Restriction for Weight				Pressure to Eat	
			**Boys**		**Girls**		**Boys**		**Girls**			
			**β [95% CI]**	***p***	**β [95% CI]**	***p***	**β [95% CI]**	***p***	**β [95% CI]**	***p***	**β [95% CI]**	***p***
**Unadjusted model**											
	4-to-24-month appetite			0.7		<0.001		0.06		<0.001		0.02
		N	707		651		707		651		1358
		Low appetite	0.07 [−0.19; 0.34]		−0.21 [−0.44; 0.02]		−0.17 [−0.32; −0.01]		−0.12 [−0.26; 0.01]		0.15 [0.01; 0.29]
		Normal appetite	0 [Ref]		0 [Ref]		0 [Ref]		0 [Ref]		0 [Ref]
		High appetite	−0.07 [−0.29; 0.15]		0.47 [0.19; 0.76]		0.06 [−0.07; 0.20]		0.48 [0.32; 0.65]		0.15 [0.01; 0.29]
Main analyses *											
	4-to-24-month appetite			0.7		<0.001		0.06		<0.001		0.03
		N	707		651		707		651		1358
		Low appetite	0.06 [−0.21; 0.33]		−0.22 [−0.46; 0.01]		−0.17 [−0.32; −0.01]		−0.12 [−0.25; 0.01]		0.15 [0.01; 0.28]
		Normal appetite	0 [Ref]		0 [Ref]		0 [Ref]		0 [Ref]		0 [Ref]
		High appetite	−0.07 [−0.29; 0.16]		0.44 [0.16; 0.72]		0.07 [−0.07; 0.20]		0.46 [0.30; 0.62]		0.14 [0.00; 0.28]
Sensitivity analyses *											
	4-to-24-month appetite, without children born preterm			0.5		0.001		0.1		<0.001		0.03
		N	667		617		667		617		1284
		Low appetite	0.07 [−0.20; 0.35]		−0.13 [−0.37; 0.11]		−0.16 [−0.33; 0.00]		−0.11 [−0.25; 0.02]		0.16 [0.01; 0.30]
		Normal appetite	0 [Ref]		0 [Ref]		0 [Ref]		0 [Ref]		0 [Ref]
		High appetite	−0.12 [−0.36; 0.12]		0.50 [0.21; 0.78]		0.01 [−0.13; 0.15]		0.49 [0.33; 0.65]		0.14 [−0.01; 0.28]
	4-to-24-month appetite, further adjusted for WHO weight-for-length z-score			0.7		0.009		0.5		<0.001		0.02
		N	707		651		707		651		1358
		Low appetite	0.06 [−0.21; 0.33]		−0.17 [−0.41; 0.06]		−0.09 [−0.25; 0.07]		−0.06 [−0.20; 0.07]		0.10 [−0.04; 0.24]
		Normal appetite	0 [Ref]		0 [Ref]		0 [Ref]		0 [Ref]		0 [Ref]
		High appetite	−0.07 [−0.29; 0.16]		0.38 [0.09; 0.67]		0.00 [−0.13; 0.13]		0.40 [0.24; 0.56]		0.19 [0.05; 0.33]
	4-to-12-month appetite			0.5		0.001		0.09		0.04		0.2
		N	707		651		707		651		1358
		Low appetite	−0.05 [−0.40; 0.31]		−0.36 [−0.63; −0.09]		−0.24 [−0.45; −0.03]		−0.09 [−0.25; 0.06]		0.01 [−0.16; 0.18]
		Normal appetite	0 [Ref]		0 [Ref]		0 [Ref]		0 [Ref]		0 [Ref]
		High appetite	−0.15 [−0.41; 0.11]		0.46 [0.10; 0.82]		−0.04 [−0.19; 0.12]		0.32 [0.11; 0.52]		0.15 [−0.02; 0.32]
	4-to-24-month appetite, multiple imputation			0.8		0.001		0.04		<0.001		0.04
		N	729		671		729		671		1400
		Low appetite	0.04 [−0.23; 0.31]		−0.22 [−0.45; 0.02]		−0.17 [−0.33; −0.01]		−0.11 [−0.24; 0.02]		0.13 [0.00; 0.27]
		Normal appetite	0 [Ref]		0 [Ref]		0 [Ref]		0 [Ref]		0 [Ref]
		High appetite	−0.07 [−0.30; 0.15]		0.43 [0.15; 0.72]		0.07 [−0.06; 0.20]		0.45 [0.29; 0.61]		0.13 [−0.01; 0.27]	

One model per exposition variable. Data are β [95% confidence intervals]. * Linear regression analyses adjusted for study center, maternal age at delivery, primiparity, maternal education level, household income, smoking status during pregnancy, child’s sex—when analyses were not stratified on child’s sex, birth weight, gestational age, prematurity and any breastfeeding duration. The interaction between child’s sex and infant’s appetite was tested for each parental feeding practices and conducted to a stratification on child’s sex for restriction for health (*p*_for interaction_ = 0.003) and restriction for weight (*p*_for interaction_ = 0.0004) but not for parental pressure to eat (*p*_for interaction_ = 0.3).

**Table 4:** Association between infant’s appetite (reference = normal appetite) and parental feeding practices of using food to regulate child’s emotions.

			Emotional Feeding (Ref = Normal)			
			**Boys**		**Girls**	
			**High**	***p***	**High**	***p***
**Unadjusted Model**						
	4-to-24-month appetite			0.002		0.5
		*N*	707		651	
		Low appetite	1.47 [0.85; 2.53]		0.79 [0.51; 1.25]	
		Normal appetite	1 [Ref]		1 [Ref]	
		High appetite	2.29 [1.41; 3.72]		1.20 [0.68; 2.11]	
Main analyses *						
	4-to-24-month appetite			0.004		0.5
		*N*	707		651	
		Low appetite	1.48 [0.85; 2.58]		0.79 [0.50; 1.27]	
		Normal appetite	1 [Ref]		1 [Ref]	
		High appetite	2.24 [1.36; 3.68]		1.13 [0.63; 2.02]	
Sensitivity analyses *						
	4-to-24-month appetite, without children born preterm			0.006		0.7
		*N*	667		617	
		Low appetite	1.49 [0.84; 2.64]		0.82 [0.50; 1.35]	
		Normal appetite	1 [Ref]		1 [Ref]	
		High appetite	2.26 [1.33; 3.85]		1.05 [0.58; 1.89]	
	4-to-24-month appetite, further adjusted for WHO weight-for-length z-score			0.005		0.7
		*N*	707		651	
		Low appetite	1.54 [0.88; 2.72]		0.83 [0.52; 1.35]	
		Normal appetite	1 [Ref]		1 [Ref]	
		High appetite	2.16 [1.31; 3.57]		1.06 [0.59; 1.91]	
	4-to-12-month appetite			0.02		0.5
		*N*	707		651	
		Low appetite	1.40 [0.66; 2.95]		0.79 [0.46; 1.35]	
		Normal appetite	1 [Ref]		1 [Ref]	
		High appetite	2.32 [1.28; 4.22]		1.29 [0.61; 2.71]	
	4-to-24-month appetite, multiple imputation			0.006		0.5
		*N*	729		671	
		Low appetite	1.04 [0.70; 1.53]		0.81 [0.58; 1.14]	
		Normal appetite	1 [Ref]		1 [Ref]	
		High appetite	1.41 [0.99; 2.01]		1.14 [0.77; 1.69]	

One model per exposition variable. Data are odds ratios [95% confidence intervals]. * Logistic regression analyses adjusted for a study center, maternal age at delivery, primiparity, maternal education level, household income, smoking status during pregnancy, birth weight, gestational age, prematurity and any breastfeeding duration. The interaction between child’s sex and infant’s appetite was tested for each parental feeding practices and conducted to a stratification on child’s sex for emotional feeding (*p*_for interaction_ = 0.02).

**Table 5:** Associations between child’s genetic susceptibility to obesity and coercive feeding practices.

		Restriction for Health		Restriction for Weight		Pressure to Eat	
		β [95% CI]	*p*	β [95% CI]	*p*	β [95% CI]	*p*
**Unadjusted model**						
	Child BMI-GRS, per risk allele (*n* = 932)	−0.01 [−0.03; 0.02]	0.6	0.01 [−0.01; 0.02]	0.3	0.01 [−0.01; 0.03]	0.3
Main analyses *						
	Child BMI-GRS, per risk allele (*n* = 932)	−0.01 [−0.03; 0.02]	0.7	0.01 [−0.01; 0.02]	0.3	0.01 [−0.01; 0.03]	0.3
Sensitivity analyses *						
	Child weighted BMI-GRS, per risk allele (*n* = 932)	−0.01 [−0.03; 0.01]	0.5	0.01 [−0.01; 0.02]	0.4	0.01 [−0.01; 0.02]	0.6
	Child BMI-GRS without children born preterm, per risk allele (*n* = 894)	−0.01 [−0.03; 0.02]	0.7	0.01 [−0.01; 0.03]	0.2	0.01 [−0.01; 0.03]	0.4
	Child BMI-GRS, per risk allele, after multiple imputation (*n* = 1342) ^a^	0.00 [−0.03; 0.02]	0.7	0.01 [−0.01; 0.02]	0.3	0.02 [0.00; 0.03]	0.08

One model per exposition variable. Data are β [95% confidence intervals]. * Linear regression analyses adjusted for study center. BMI-GRS, genetic risk score of body mass index. ^a^ Missing data for child’s BMI-GRS were only imputed if maternal BMI-GRS was available.

**Table 6:** Associations between child’s genetic susceptibility to obesity and parental feeding practices of using food for non-nutritional purposes.

		Emotional Feeding	
		OR [95% CI]	*p*
**Unadjusted model**		
	Child BMI-GRS, per risk allele (*n* = 932)	1.00 [0.95; 1.05]	1
Main analyses *		
	Child BMI-GRS, per risk allele (*n* = 932)	1.00 [0.95; 1.05]	0.9
Sensitivity analyses *		
	Child weighted BMI-GRS, per risk allele (*n* = 932)	1.01 [0.97; 1.06]	0.6
	Child BMI-GRS without children born preterm, per risk allele (*n* = 894)	1.01 [0.95; 1.06]	0.8
	Child BMI-GRS, per risk allele, after multiple imputation (*n* = 1342) ^a^	1.00 [0.95; 1.04]	0.9

One model per exposition variable. Data are odds ratios [95% confidence intervals]. * Logistic regression analyses adjusted for study center. BMI-GRS, genetic risk score of body mass index. ^a^ Missing data for child’s BMI-GRS were only imputed if maternal BMI-GRS was available.

## Data Availability

The data underlying the findings cannot be made freely available for ethical and legal restrictions imposed because this study includes a substantial number of variables that, together, could be used to re-identify the participants based on a few key characteristics and then be used to have access to other personal data. Therefore, the French ethics authority strictly forbids making these data freely available. However, they can be obtained upon request from the EDEN principal investigator. Readers may contact barbara.heude@inserm.fr to request the data. The analytic code will be made available upon request pending application and approval.

## Supplementary Materials

The following are available online at https://www.mdpi.com/article/10.3390/nu13051468/s1, Table S1: Details regarding multiple imputations. Table S2: Sensitivity analyses: associations between infant’s appetite (reference = normal appetite) and parental feeding practice of using food as a reward. Table S3: Sensitivity analyses: associations between child’s genetic susceptibility to obesity and parental feeding practice of using food as a reward. Table S4: Sensitivity analyses: associations between child’s appetite (reference = normal appetite) at 1 year and coercive parental feeding practices. Table S5: Sensitivity analyses: associations between child’s appetite (reference = normal appetite) at 1 year and parental feeding practices of using food for non-nutritional purposes. Table S6: STROBE statement.
