# Supplementary material for: Associations between Children’s Genetic Susceptibility to Obesity, Infant’s Appetite and Parental Feeding Practices in Toddlerhood
Source: Nutrients. 2021 Apr 26;13(5):1468. doi: 10.3390/nu13051468 (PMC8146167; doi:10.3390/nu13051468)
Supplement: Supplementary file 1 [file nutrients-13-01468-s001.zip › nutrients-1155827-supplementary.pdf]

Supplemental Table S1. Details regarding multiple imputations

Variable	Type of variable	Model used to predict missing data	Missing values
Restriction for health	Continuous	No missing data	0%
Restriction for weight	Continuous	No missing data	0%
Pressure to eat	Continuous	No missing data	0%
Food as a reward	Continuous	No missing data	0%
Emotional feeding	Continuous	No missing data	0%
Study center	Binary	No missing data	0%
Maternal age at delivery	Continuous	No missing data	0%
Child sex	Binary	No missing data	0%
Preterm birth	Binary	No missing data	0%
Birth weight	Continuous	No missing data	0%
Indicator of early appetitive traits	Categorical (3 categories)	No missing data	0%
Any breastfeeding duration	Categorical (3 categories)	Logistic regression	1 (0.07%)
Age at complementary food introduction	Continuous	Linear regression (ppm)	1 (0.07%)
Appetite at 2 years	Categorical (3 categories)	Logistic regression	1 (0.07%)
Primiparity	Binary	Logistic regression	2 (0.1%)
Maternal education level	Categorical (4 categories)	Logistic regression	7 (0.5%)
Household income	Categorical (4 categories)	Logistic regression	9 (0.6%)
Smoking status during pregnancy	Binary	Logistic regression	31 (2.2%)
Appetite at 4 months	Categorical (3 categories)	Logistic regression	34 (2.4%)
Appetite at 8 months	Categorical (3 categories)	Logistic regression	46 (3.3%)
Appetite at 1 year	Categorical (3 categories)	Logistic regression	72 (5.1%)
Child BMI-GRS	Continuous	Linear regression (ppm)	435 (31.1%)

BMI-GRS, genetic risk score of body mass index

Supplemental Table S2. Sensitivity analyses: associations between infant's appetite (reference = normal appetite) and parental feeding practice of using food as a reward

	Food as a reward (Ref=Normal)			
	Boys		Girls	
	High	P	High	P
**Unadjusted model**				
4-to-24-month appetite		<0.001		0.002
N	707		651	
Low appetite	2.67 [1.51; 4.69]		0.54 [0.34; 0.85]	
Normal appetite	1 [Ref]		1 [Ref]	
High appetite	1.65 [1.06; 2.56]		1.83 [1.02; 3.29]	
**Main analyses ***				
4-to-24-month appetite		0.001		0.007
N	707		651	
Low appetite	2.69 [1.50; 4.81]		0.53 [0.33; 0.86]	
Normal appetite	1 [Ref]		1 [Ref]	
High appetite	1.58 [1.01; 2.49]		1.64 [0.89; 3.01]	
**Sensitivity analyses ***				
4-to-24-month appetite, without children born preterm		0.005		0.02
N	667		617	
Low appetite	2.43 [1.35; 4.38]		0.58 [0.35; 0.97]	
Normal appetite	1 [Ref]		1 [Ref]	
High appetite	1.54 [0.95; 2.49]		1.72 [0.92; 3.20]	
4-to-24-month appetite, further adjusted for WHO weight-for-length z-score		0.001		0.008
N	707		651	
Low appetite	2.58 [1.43; 4.64]		0.53 [0.32; 0.86]	
Normal appetite	1 [Ref]		1 [Ref]	
High appetite	1.64 [1.04; 2.60]		1.65 [0.89; 3.07]	
4-to-12-month appetite		0.07		0.06
N	707		651	
Low appetite	1.93 [0.92; 4.09]		0.63 [0.36; 1.10]	
Normal appetite	1 [Ref]		1 [Ref]	
High appetite	1.55 [0.91; 2.63]		1.89 [0.86; 4.15]	
4-to-24-month appetite, multiple imputation		0.001		0.004
N	729		671	
Low appetite	1.63 [1.10; 2.41]		0.55 [0.38; 0.78]	
Normal appetite	1 [Ref]		1 [Ref]	
High appetite	0.98 [0.70; 1.37]		1.70 [1.13; 2.57]	

One model per exposition variable.
Data are odds ratios [95% confidence intervals].
* Logistic regression analyses adjusted for [a] study center, maternal age at delivery, primiparity, maternal education level, household income, smoking status during pregnancy, birth weight, gestational age, prematurity and any breastfeeding duration.
The interaction between child's sex and infant's appetite was tested for each parental feeding practices and conducted to a stratification on child's sex for food as a reward ($p_{\text{for interaction}}$=0.08).

Supplemental Table S3. Sensitivity analyses: associations between child's genetic susceptibility to obesity and parental feeding practice of using food as a reward

	Food as a reward	
	OR [95% CI]	P
**Unadjusted model**		
Child BMI-GRS, per risk allele (n=932)	1.03 [0.98; 1.08]	0.3
**Main analyses ***		
Child BMI-GRS, per risk allele (n=932)	1.03 [0.98; 1.09]	0.3
**Sensitivity analyses ***		
Child weighted BMI-GRS, per risk allele (n=932)	1.02 [0.98; 1.07]	0.4
Child BMI-GRS without children born preterm, per risk allele (n=894)	1.02 [0.97; 1.08]	0.4
Child BMI-GRS, per risk allele, after multiple imputation (n=1342) [a]	1.02 [0.97; 1.07]	0.2

One model per exposition variable.

Data are odds ratios [95% confidence intervals].

* Logistic regression analyses adjusted for study center.

BMI-GRS, genetic risk score of body mass index.

[a] Missing data for child BMI-GRS were only imputed if maternal BMI-GRS was available.

Supplemental Table S4. Sensitivity analyses: associations between child's appetite (reference = normal appetite) at 1 year and coercive parental feeding practices

	Restriction for health				Restriction for weight				Pressure to eat	
	Boys		Girls		Boys		Girls			
	β [95% CI]	P	β [95% CI]	P	β [95% CI]	P	β [95% CI]	P	β [95% CI]	P
Child appetite at 1 year		0.3		0.03		0.03		0.04		0.8
N	682		610		682		610		1292	
Low appetite	-0.29 [-0.76; 0.17]		-0.42 [-0.74; -0.10]		-0.38 [-0.65; -0.10]		-0.16 [-0.34; 0.03]		0.00 [-0.21; 0.20]	
Normal appetite	0 [Ref]		0 [Ref]		0 [Ref]		0 [Ref]		0 [Ref]	
High appetite	-0.18 [-0.56; 0.21]		0.19 [-0.36; 0.74]		0.02 [-0.21; 0.24]		0.31 [-0.01; 0.63]		0.08 [-0.17; 0.33]	

Data are β [95% confidence intervals].

Linear regressions adjusted for study center, maternal age at delivery, primiparity, maternal education level, household income, smoking status during pregnancy, child sex - when analyses were not stratified on child sex-, birth weight, gestational age, prematurity and any breastfeeding duration.

Supplemental Table S5. Sensitivity analyses: associations between child's appetite (reference = normal appetite) at 1 year and other parental feeding practices

	Food as a reward (Ref=Normal)				Emotional feeding (Ref=Normal)			
	Boys		Girls		Boys		Girls	
	High	P	High	P	High	P	High	P
Child appetite at 1 year		0.1		0.4		0.2		0.5
N	682		610		682		610	
Low appetite	1.70 [0.64; 4.51]		0.66 [0.35; 1.28]		1.05 [0.41; 2.69]		0.70 [0.37; 1.33]	
Normal appetite	1 [Ref]		1 [Ref]		1 [Ref]		1 [Ref]	
High appetite	2.16 [0.94; 4.93]		1.30 [0.41; 4.14]		2.47 [0.97; 6.28]		1.37 [0.44; 4.28]	

Data are odds ratios [95% confidence intervals].

Logistic regressions adjusted for study center, maternal age at delivery, primiparity, maternal education level, household income, smoking status during pregnancy, birth weight, gestational age, prematurity and any breastfeeding duration

STROBE Statement. Checklist of items that should be included in reports of cohort studies

	Item No	Recommendation	Page No
**Title and abstract**	1	(*a*) Indicate the study's design with a commonly used term in the title or the abstract	Title page
		(*b*) Provide in the abstract an informative and balanced summary of what was done and what was found	Title page
**Introduction**			
Background/rationale	2	Explain the scientific background and rationale for the investigation being reported	Introduction, paragraphs 1-2
Objectives	3	State specific objectives, including any prespecified hypotheses	Introduction, paragraph 3
**Methods**			
Study design	4	Present key elements of study design early in the paper	Materials and methods, paragraph 2.1
Setting	5	Describe the setting, locations, and relevant dates, including periods of recruitment, exposure, follow-up, and data collection	Materials and methods, paragraph 2.1
Participants	6	(*a*) Give the eligibility criteria, and the sources and methods of selection of participants. Describe methods of follow-up<br>(*b*) For matched studies, give matching criteria and number of exposed and unexposed	Materials and methods, paragraph 2.6
Variables	7	Clearly define all outcomes, exposures, predictors, potential confounders, and effect modifiers. Give diagnostic criteria, if applicable	Materials and methods, paragraphs 2.2, 2.3, 2.4 and 2.5
Data sources/ measurement	8*	For each variable of interest, give sources of data and details of methods of assessment (measurement). Describe comparability of assessment methods if there is more than one group	Materials and methods, paragraphs 2.2, 2.3, 2.4 and 2.5
Bias	9	Describe any efforts to address potential sources of bias	
Study size	10	Explain how the study size was arrived at	Materials and methods, paragraph 2.6
Quantitative variables	11	Explain how quantitative variables were handled in the analyses. If applicable, describe which groupings were chosen and why	Materials and methods, paragraphs 2.2, 2.3, 2.4 and 2.5
Statistical methods	12	(*a*) Describe all statistical methods, including those used to control for confounding<br>(*b*) Describe any methods used to examine subgroups and interactions<br>(*c*) Explain how missing data were addressed<br>(*d*) If applicable, explain how loss to follow-up was addressed<br>(*e*) Describe any sensitivity analyses	Materials and methods, paragraph 2.7
**Results**			
Participants	13*	(a) Report numbers of individuals at each stage of study—eg numbers potentially eligible, examined for eligibility, confirmed eligible, included in the study, completing follow-up, and analysed<br>(b) Give reasons for non-participation at each stage	Figure 1

		(c) Consider use of a flow diagram	
Descriptive data	14*	(a) Give characteristics of study participants (eg demographic, clinical, social) and information on exposures and potential confounders<br><br>(b) Indicate number of participants with missing data for each variable of interest<br>(c) Summarise follow-up time (eg, average and total amount)	Results, paragraph 1<br>Tables 1 and 2
Outcome data	15*	Report numbers of outcome events or summary measures over time	Tables 1 and 2
Main results	16	(*a*) Give unadjusted estimates and, if applicable, confounder-adjusted estimates and their precision (eg, 95% confidence interval). Make clear which confounders were adjusted for and why they were included	Results, paragraph 3.1 and 3.2<br>Tables 3, to 6
		(*b*) Report category boundaries when continuous variables were categorized	
		(*c*) If relevant, consider translating estimates of relative risk into absolute risk for a meaningful time period	
Other analyses	17	Report other analyses done—eg analyses of subgroups and interactions, and sensitivity analyses	Results, paragraph 3.1 and 3.2<br>Tables 3, to 6<br>Suppl. Tables S2 to S5
**Discussion**			
Key results	18	Summarise key results with reference to study objectives	Discussion, paragraph 1
Limitations	19	Discuss limitations of the study, taking into account sources of potential bias or imprecision. Discuss both direction and magnitude of any potential bias	Discussion, paragraph 5
Interpretation	20	Give a cautious overall interpretation of results considering objectives, limitations, multiplicity of analyses, results from similar studies, and other relevant evidence	Discussion, paragraphs 2 to 4
Generalisability	21	Discuss the generalisability (external validity) of the study results	Discussion, paragraph 5
Other information			
Funding	22	Give the source of funding and the role of the funders for the present study and, if applicable, for the original study on which the present article is based	Funding section

*Give information separately for exposed and unexposed groups.

Note: An Explanation and Elaboration article discusses each checklist item and gives methodological background and published examples of transparent reporting. The STROBE checklist is best used in conjunction with this article (freely available on the Web sites of PLoS Medicine at http://www.plosmedicine.org/, Annals of Internal Medicine at http://www.annals.org/, and Epidemiology at http://www.epidem.com/). Information on the STROBE Initiative is available at http://www.strobe-statement.org.
